# Conversion of Fused Knee Following Distal Femur Tumor Surgery to Total Knee Arthroplasty

**DOI:** 10.5812/ircmj.7693

**Published:** 2013-09-05

**Authors:** Khodamorad Jamshidi, Hoseinali Hadi, Mehdi Ramezan Shirazi, Alireza Moslem

**Affiliations:** 1 Department of Orthopedic Surgery, Iran University of Medical Sciences, Tehran, IR Iran; 2 Department of Orthopedic Surgery, Arak University of Medical Science, Arak, IR Iran; 3 Department of Anesthesiology, Gonabad University of Medical Sciences, Gonabad, IR Iran; 4 Department of Orthopedic Surgeon, Gonabad University of Medical Sciences, Gonabad, IR Iran

**Keywords:** Knee, Arthrodesis, Osteosarcoma, Arthroplasty, Replacement, Knee

## Abstract

Conversion of knee arthrodesis to total knee arthroplasty is a difficult procedure accompanied by many complications due to soft tissue and extensor mechanism contracture and bone defects. We report two cases of distal femur osteosarcoma that had been undergone wide resection arthrodesis initially. Arthrodesis was converted to total knee arthroplasty with hinged prosthesis after many years. We describe patients' history and outline their surgical therapy and prognosis. To the best of the authors' knowledge, few cases have been previously reported in the literature.

## 1. Introduction

Total knee arthroplasty techniques are successfully applied to increasing challenging reconstructive problems. In contrast to many reports in hip pathology which confirm the effectiveness of total hip arthroplasty in previous hip fusion (1, 2), conversion of fused knee to total knee arthroplasty is a very difficult procedure and few cases have been reported in the literature. Results of knee arthroplasty after spontaneous ankylosis and knee arthrodesis can be different since soft tissue of the knee may be disrupted after previous multiple surgeries (3). Soft tissue and extensor mechanisms are main factors in achieving good results after conversion of fused knee to arthroplasty. When the skin is in poor condition, the patient might benefit from a skin distension procedure or a local or micro-vascular free flap as a preliminary step prior to total knee arthroplasty (4).

Collateral ligaments balancing after take down of the knee fusion is difficult and often leads to instability. Therefore, hinged or condylar constrain prostheses are classically recommended in these situations (5, 6). We report two cases of distal femur osteosarcoma that had been initially undergone wide resection and arthrodesis and then were converted to total knee arthroplasty with hinged prosthesis after many years. 

## 2. Case Reports

### 2.1. Case 1

**Figure 1.**
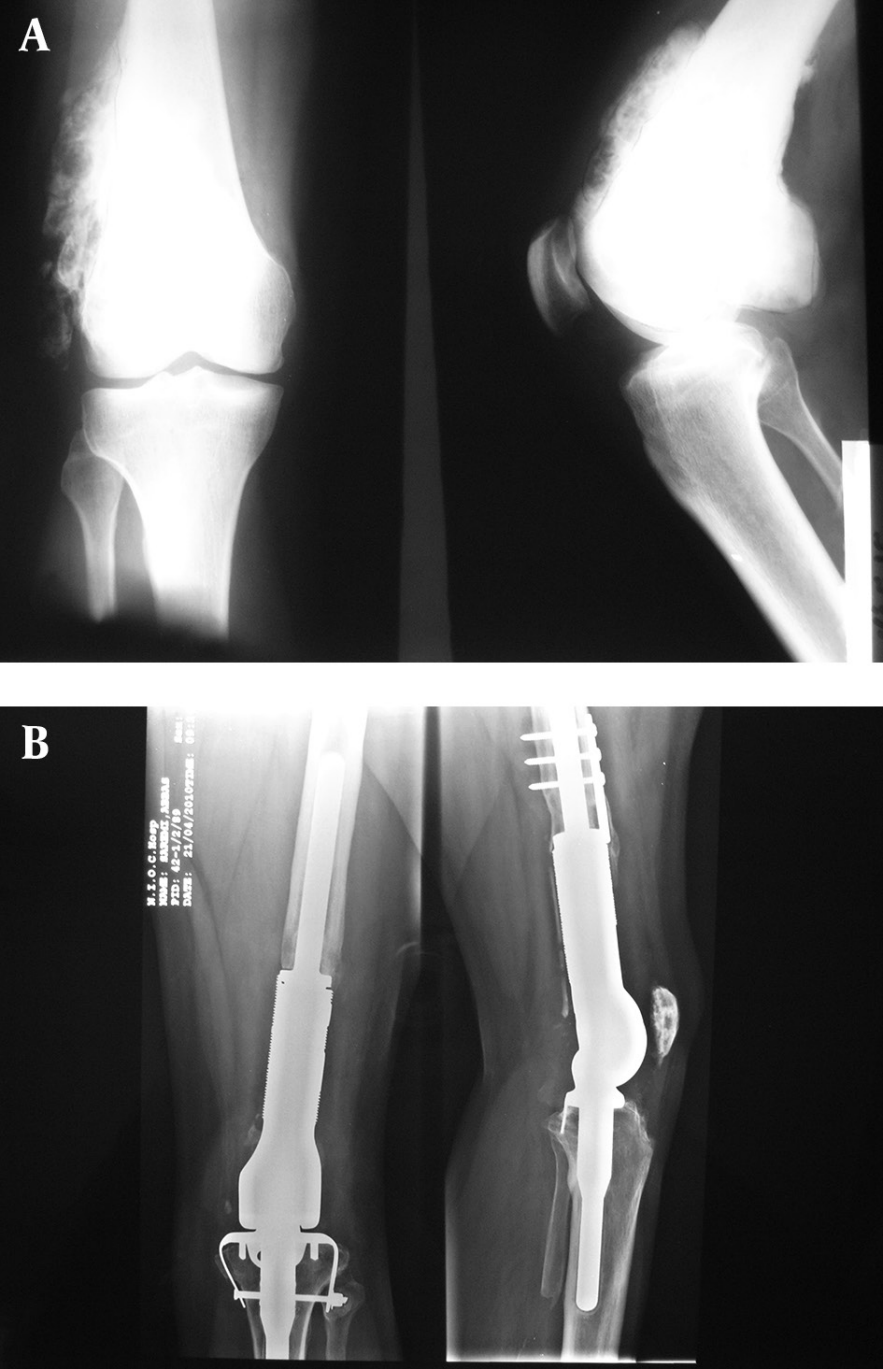
Anteroposterior and Lateral X-rays of the Left Knee Showing Distal Femur Parosteal Osteosarcoma (A). Post Total Knee Arthroplasty X-rays After Knee Fusion (B).

A 40-year old man first presented a left distal femur parosteal osteosarcoma 15 years ago. Wide resection and knee arthrodesis with ipsilateral fibular autograft and internal fixation with intramedullary nail and pins had been undergone (Figure 1). Fracture was developed in the site of arthrodesis 4 years ago and lead to nonunion due to device failure. Conversion of artherodesis to arthroplasty with all probable complications was recommended to the patient. He was treated with a left total knee arthroplasty with a HMRS (Howmedica Modular Replacement System, Stryker) prosthesis with patellar resurfacing (Figure 1). The initial bony cuts were made through the fusion site. 

During the early postoperative period, no significant complications were happened. He was ambulatory with the aid of support on the fourth day. Function was improved substantially. Postoperatively, anteroposterior and lateral plain x – ray were made regularly. Accordingly, there were no signs of prosthesis loosening. The patient was examined by the senior author at all follow-ups. Particular attention was paid to any evidence of instability. At the time of the most recent follow up, the knee stability was excellent. He was pain free and walked without any support and brace. His left knee range of motion was 0-100 degree and his quadriceps muscle strength 5.5. Knee Society Score increased from 80 to 85. The patient complained of buckling sensation only occasionally while walking. Based on British orthopedic society score, the patient had the maximum level of satisfaction (point 4 or enthusiastic). 

### 2.2. Case 2

**Figure 2.**
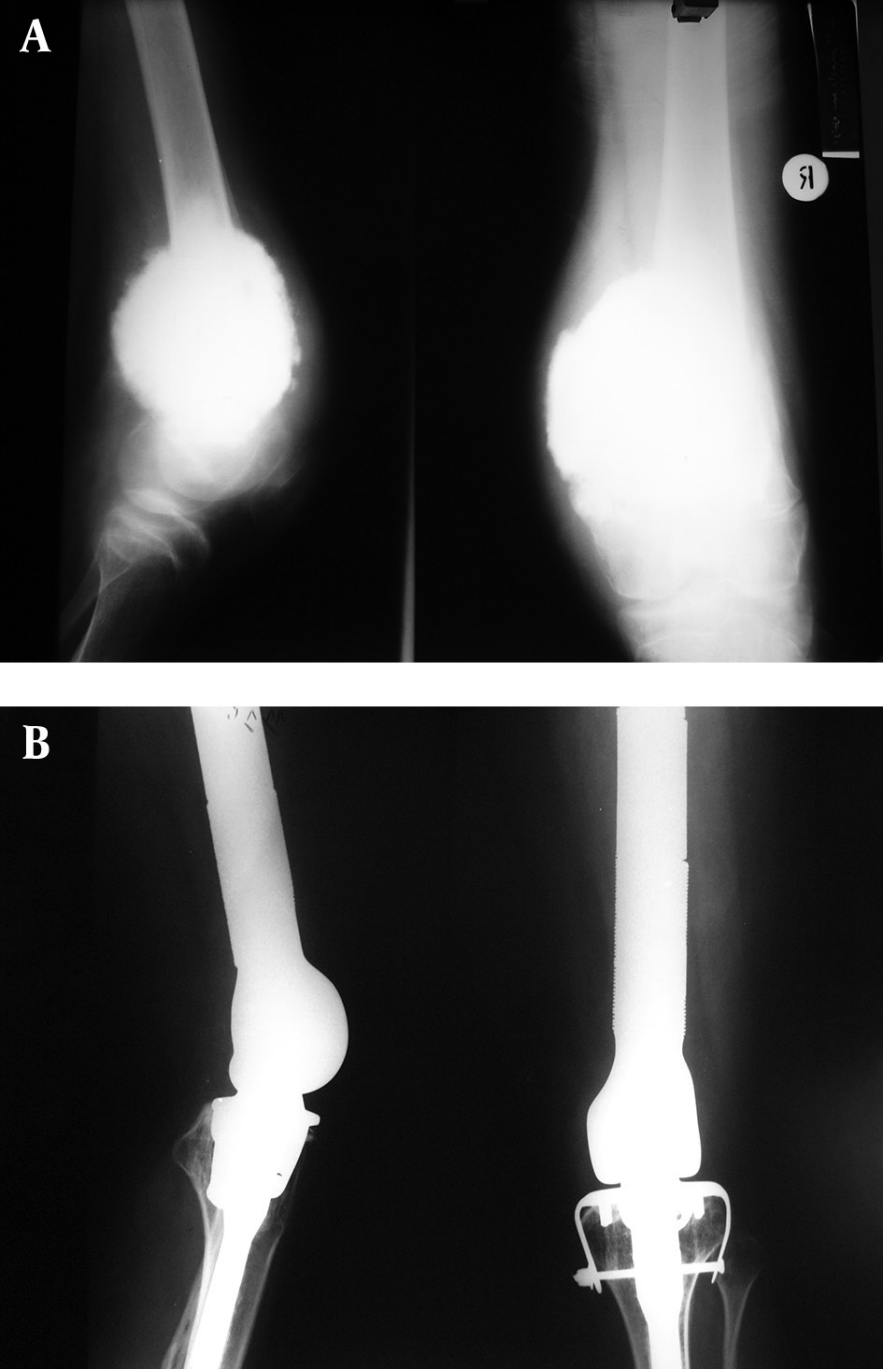
Anteroposterior and Lateral X-rays of the Right Knee Showing Distal Femur Conventional Osteosarcoma (A). Post Total Knee Arthroplasty X-rays After Knee Fusion (B).

A 27-year old woman visited our clinic with a conventional osteosarcoma of right distal femur 14 years ago. Surgery including wide resection and knee arthrodesis with ipsilateral fibular autograft and fixation with interamedullary nail and pins was performed (Figure 2). Fracture and subsequent nonunion on arthrodesis site occurred 18 month ago. Right total knee arthroplasty with HMRS prosthesis was performed. Postoperatively, there were no significant complications except skin edge necrosis and serous discharge treated with debridement and wound closure. She was ambulatory with a support on the fifth day. She has functioned reasonably well since then. The patient was examined by the senior author at all follow-ups. Particular attention was paid to any evidence of instability. At the last follow-up, she had no pain, deformity and instability. She walked without support. Her knee range of motion was 0-120 degrees. In spite of hamstring transfer in the second surgery, quadriceps strength was 1.5 and had 15 degrees extension lag due to quadriceps muscle excision at the first surgery. Knee society score increased from 65 preoperatively to 85 postoperatively and functional score increased from 85 to 90. Based on British orthopedic society score, patient satisfaction is 3 (satisfied) out of 4 points. 

## 3. Discussion

In contrast to hip arthrodesis, knee arthrodesis is known as an irreversible procedure. Patients with knee arthrodesis or ankylosis have many psychological problems and functional disabilities that need to be considered. Normal walking and sitting are very important for normal sensation (7). Extensive bone defects and ligament instability after knee fusion take down are difficult to manage with usual low constrain prostheses. These problems are better managed with high constrain (e.g. rotary hinge) prosthesis. Although hinge prostheses provide enough stability, they are accompanied with a high incidence of loosening and infection (7). Kim et al. performed total knee arthroplasty and postoperative traction on 27 knees (24 patients) with spontaneous bony ankylosis in severe knee flexion. Preoperative Hospital for Special Surgery knee score of 60 increased to 87 postoperatively. On an average 4.6 years of follow-up, no patients had needed revision for prosthetic loosening (8).

Irwin et al. studied the function of three groups of patients who had a malignant skeletal tumor around the knee that had undergone resection arthrodesis, above the knee amputation, and replacement arthroplasty. In this study, patients’ function was similar in all groups. Patients who had had an arthrodesis had a more stable limb and performed most physical and recreational activities, but had difficulty sitting. Patients who had had an arthroplasty had sedentary and low demand lives and were more protective of the limb, but they were the least self-conscious group about the limb (9). With the ability to take down fusions of long standing duration as a viable alternative, the importance of saving as much bone as possible , especially patella bone stock in cases of knee fusion cannot be overemphasized. In fused knee cases that have good bone stock, owing to capsular contracture in medial and lateral, enough stability for the use of usual prostheses can be achieved, but higher constrain prostheses should always be available (10).

In these two cases, the patients’ walking ability and arthrodesis stability had decreased because of arthrodesis site fracture and nail failure; therefore, conversion to arthroplasty was recommended. Because of severe bone and soft tissue loss, modular replacement hinged prosthesis was applied, so there was no concern over ligament stability. Bony cut was made through the junction of the normal bone and the graft due to following reasons:

1.Inserting the femoral stem into the normal intramedullary canal was desired

2.Nonvascularized fibular graft was thought not to be viable.

Skin edge necrosis and serous discharge from surgical wound were observed in case 2 . This complication was treated with surgical debridement and wound closure. In this case, there was also severe extension lag because of extensor mechanism and patellar excision during initial wide resection for tumor surgery. There was no evidence of infection or loosening in clinical and radiographic assessment in the last follow-up. In both cases, increase in the range of motion and improvement in sitting and walking ability led to patients’ satisfaction. Although duration of follow-up was short and more cases are required to make more comprehensive suggestions, conversion of arthrodesis to arthroplasty can be considered as a good option in patients whose arthrodesis has been problematic.

Anterior knee soft tissue stiffness and quadriceps contracture made the conversion of fused knee to total knee arthroplasty very difficult. Bone defects and ligament instability added to the problems. A well-planned arthroplasty with full range of implants and instruments available, and attention to soft tissue and extensor mechanisms can produce successful results even in very difficult situations such as these two cases. Conversion of a fused knee to arthroplasty should be done at a center with sufficient equipment and by an experienced surgeon in knee arthroplasty. However, patient’s understanding and expectations about the results and complications is of prime importance. This procedure can have its place in orthopedic surgeon armamentarium, if above problems are taken into consideration.
